# Facial-Aging Mobile Apps for Smoking Prevention in Secondary Schools in Brazil: Appearance-Focused Interventional Study

**DOI:** 10.2196/10234

**Published:** 2018-07-17

**Authors:** Breno Bernardes-Souza, Francisco Patruz Ananias De Assis Pires, Gustavo Moreira Madeira, Túlio Felício Da Cunha Rodrigues, Martina Gatzka, Markus V Heppt, Albert J Omlor, Alexander H Enk, David A Groneberg, Werner Seeger, Christof von Kalle, Carola Berking, Paulo César Rodrigues Pinto Corrêa, Janina Leonie Suhre, Jonas Alfitian, Aisllan Assis, Titus Josef Brinker

**Affiliations:** ^1^ School of Medicine Federal University of Ouro Preto Ouro Preto Brazil; ^2^ University of Ulm Department of Dermatology and Allergic Diseases Ulm Germany; ^3^ University Medical Center Munich Department of Dermatology and Allergology Munich Germany; ^4^ Saarland University Medical Center Department of Experimental Pneumology and Allergology Saarland University Homburg Germany; ^5^ Heidelberg University Hospital Department of Dermatology University of Heidelberg Heidelberg, Germany Germany; ^6^ Institute of Occupational Medicine, Social Medicine and Environmental Medicine Goethe-University of Frankfurt Frankfurt Germany; ^7^ Excellence Cluster Cardiopulmonary System, University of Giessen and Marburg Lung Center (UGMLC), member of the German Center for Lung Research (DZL) Justus-Liebig-University Gießen Germany; ^8^ National Center for Tumor Diseases (NCT) Department of Translational Oncology German Cancer Research Center Heidelberg Germany; ^9^ University Hospital of Bonn Department of Pulmonary Medicine University of Bonn Bonn Germany; ^10^ University Hospital of Cologne Department of Cardiology University of Cologne Cologne Germany; ^11^ German Cancer Consortium (DKTK) University of Heidelberg Heidelberg Germany

**Keywords:** dermatology, smoking, apps, photoaging, face, skin, tobacco, tobacco cessation, tobacco prevention

## Abstract

**Background:**

Most smokers start smoking during their early adolescence, often with the idea that smoking is glamorous. Interventions that harness the broad availability of mobile phones as well as adolescents' interest in their appearance may be a novel way to improve school-based prevention. A recent study conducted in Germany showed promising results. However, the transfer to other cultural contexts, effects on different genders, and implementability remains unknown.

**Objective:**

In this observational study, we aimed to test the perception and implementability of facial-aging apps to prevent smoking in secondary schools in Brazil in accordance with the theory of planned behavior and with respect to different genders.

**Methods:**

We used a free facial-aging mobile phone app (“Smokerface”) in three Brazilian secondary schools via a novel method called mirroring. The students’ altered three-dimensional selfies on mobile phones or tablets and images were “mirrored” via a projector in front of their whole grade. Using an anonymous questionnaire, we then measured on a 5-point Likert scale the perceptions of the intervention among 306 Brazilian secondary school students of both genders in the seventh grade (average age 12.97 years). A second questionnaire captured perceptions of medical students who conducted the intervention and its conduction per protocol.

**Results:**

The majority of students perceived the intervention as fun (304/306, 99.3%), claimed the intervention motivated them not to smoke (289/306, 94.4%), and stated that they learned new benefits of not smoking (300/306, 98.0%). Only a minority of students disagreed or fully disagreed that they learned new benefits of nonsmoking (4/306, 1.3%) or that they themselves were motivated not to smoke (5/306, 1.6%). All of the protocol was delivered by volunteer medical students.

**Conclusions:**

Our data indicate the potential for facial-aging interventions to reduce smoking prevalence in Brazilian secondary schools in accordance with the theory of planned behavior. Volunteer medical students enjoyed the intervention and are capable of complete implementation per protocol.

## Introduction

### Background

Smoking is the leading global cause of preventable death, causing nearly 6 million deaths per year worldwide. A 2011 study of the tobacco-related burden in Brazil found that smoking was accountable for 147,072 deaths (403 deaths per day), 157,126 myocardial infarctions, and 63,753 cases of cancer. It generated 2.69 million disability-adjusted life years and cost the Brazilian health system US $7.37 billion in 2011 alone [1].

Most smokers start smoking during their early adolescence, often with the idea that smoking is glamorous, with the associated health consequences too far in the future to imagine. According to the Adolescent Cardiovascular Risk Study, almost 600,000 adolescents smoke regularly in Brazil and most of them tried their first cigarette between 15 and 17 years of age [2].

The earlier a person starts smoking, the higher the chance of becoming a regular smoker and developing associated diseases. As most smokers start smoking during early adolescence, it is imperative to develop, test, and validate tobacco control strategies that focus on this group through an age-appropriate and innovative approach. Most educational interventions for adolescents have focused on increasing awareness of tobacco-induced diseases [2]. These mostly fail to show sustainable effects [3].

### Research on School-Based Tobacco Prevention Interventions in Brazil

In Brazil, a 2015 randomized controlled trial at the Federal University of the State of São Paulo investigating different school-based interventions to reduce the use of various psychotropic substances among 1316 students showed mixed effects for different drugs/settings with study design limitations precluding interpretation [4].

Furthermore, a study on educational interventions among school adolescents analyzed the effectiveness of an educational program on smoking developed by the Brazilian Cancer Institute. The researchers selected 32 random schools from a total of 46 public schools in the city of Pelotas and randomized them to control and intervention schools. The total sample was 2200 students in the 7th and 8th grades (13-14 years old). They used questionnaires before and after interventions and collected urine samples in order to detect nicotine. Although the results showed no change in tobacco use reduction, they improved the students’ knowledge on passive smoking [5].

Despite these studies, data on school-based tobacco prevention interventions conducted remain scarce.

### Education Against Tobacco

Founded in Germany in 2012, Education Against Tobacco is a global network of medical students that aims to provide science-based and age-appropriate preventions to a large number of adolescents and at the same time sensitizes prospective physicians to the importance of delivering smoking cessation advice and engaging themselves in tobacco control activities after their graduation [6-10]. The network currently involves 80 medical schools in 14 countries, with 3500 medical students educating more than 50,000 secondary school students in the classroom setting per year, while using and optimizing apps and strategies. In Brazil, Education Against Tobacco was founded in 2016 and is already present in 15 medical schools in the country.

In a recent paper*,* we introduced facial-aging mobile apps that alter a person’s selfie (a self-portrait taken with a mobile phone camera) to predict future appearance if that person smokes [11]. These apps are considered a new opportunity for smoking prevention after their effectiveness was first demonstrated by Burford et al [12,13]. They are also used in other behavioral change settings, such as skin cancer prevention [14,15]. In the clinical setting, they were recently made available in waiting rooms to motivate patients to address quitting with their doctor [16] or to improve UV protection [17]. In addition to this, many dermatology publications have called for a novel public health approach in light of new findings on the facial-aging effects of smoking [18]. Facial-aging approaches indicate relevance for teenagers as evidenced by numerous publications demonstrating and investigating their influence on behavior [6,19-24]. In contrast, it is notable that the tobacco industry itself tried to establish the link between attractiveness and smoking by commercial advertising in the past [25].

We recently implemented a facial-aging mobile app (“Smokerface”) in German secondary schools via a method called mirroring [26]. We “mirrored” the students’ altered 3-dimensional (3D) selfies on mobile phones or tablets via a projector in front of their entire grade. Using an anonymous questionnaire, we then measured sociodemographic data as well as the perceptions of the intervention on a 5-point Likert scale among 125 students of both genders (average age 12.75 years). A majority of the students perceived the intervention as fun (77/125, 61.6%), claimed that the intervention motivated them not to smoke (79/125, 63.2%), and stated that they learned new benefits of nonsmoking (81/125, 64.8%).

### Theoretical Considerations on Photoaging Interventions in Adolescence

The self-concept of appearance, which photoaging interventions harness, is the strongest predictor of self-esteem in adolescents of both genders [27,28]. In the most recent publication by Baudson et al involving a sample of 2950 adolescents from a broad range of secondary schools, it was noted that this is especially true for students from lower educational schools and girls [28]. An explanation for the general effectiveness of such an intervention is given by the theory of planned behavior, according to which the subjective norm (ie, “my friends think that smoking makes you unattractive”), the attitudes (consisting of beliefs, ie, “smoking leads to unattractiveness”), and the perceived behavioral control (ie, “I can resist if somebody offers me a cigarette”) influence both the behavioral intentions of a person and their behavior. Photoaging interventions may affect all three of these predictors, and the mirroring intervention specifically had a strong influence on the subjective norm in a recent pilot study [26].

This study investigated if effects are different for female/male participants and if the results of our novel facial-aging intervention are reproducible in Brazil, a country where data on tobacco prevention programs remain scarce. Additionally, a process evaluation investigated whether local volunteering medical students are capable of complete intervention implementation.

## Methods

### Participants

We included a total sample of 306 students in Grade 7 in our cross-sectional study with an average age of 12.97 years (age range 12-16; 172/306, 56.2% female; 134/306, 43.8% male) attending three regular public secondary schools in the city of Ponte Nova in southeast Brazil (total of 15 classes). Informed consent was obtained from the parents. A large majority of participants (257/306, 84.0%) reported that they owned a smartphone.

### Setting

The mirroring approach was implemented via local medical students from the Education Against Tobacco nonprofit organization who were attending the Federal University of Ouro Preto in Brazil [7-9]. Two medical students per classroom conducted the interventions with approximately 20 students at a time (average 20.4 students, SD 4.4). To increase students’ participation in the mirroring intervention, students were encouraged to download the app (“Smokerface”) before our visit, via a letter 3 days in advance. When we visited the schools, 34.3% (105/306) of students already had the facial-aging app on their mobile phone.

### Intervention

**Figure 1:**
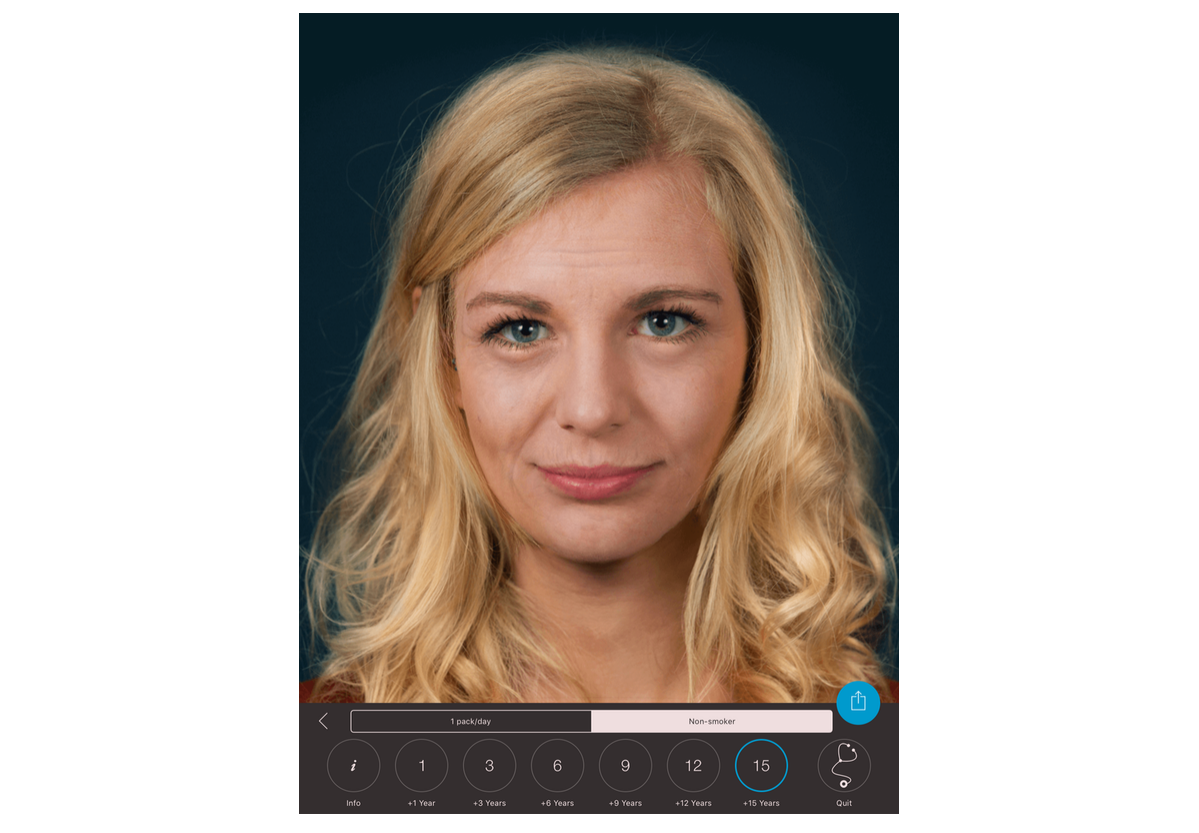
Effect view of the Smokerface app on an iOS iPad; normal aging without smoking for 15 years.

**Figure 2:**
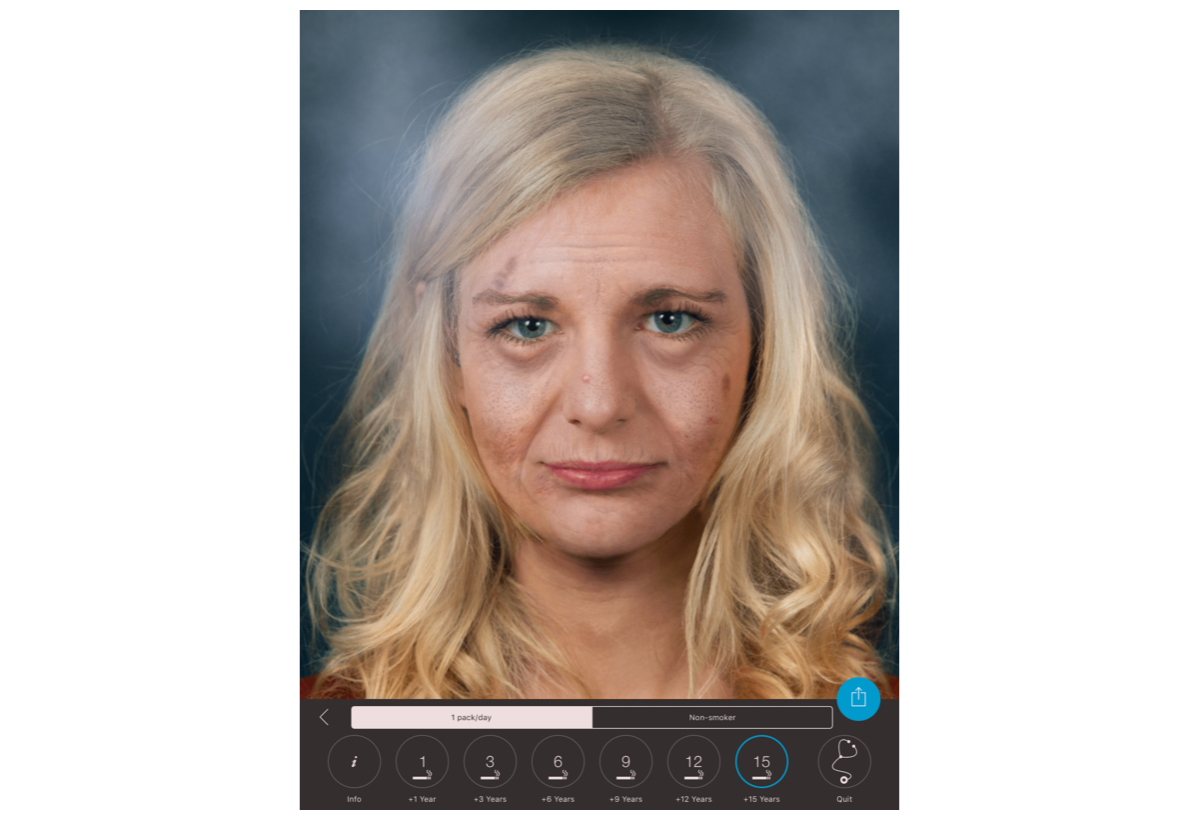
Effect view of the Smokerface app on an iOS iPad; aging with smoking one pack of cigarettes a day for 15 years.

The mirroring intervention consists of a 45-minute app-based module in the classroom setting. Mirroring means that the student’s altered 3D selfies on their mobile phones or tablets are “mirrored” via a projector in front of the whole class, for example, sneezing or coughing (Multimedia Appendix 1). In front of their peers and teachers, they could display their image as a nonsmoker/smoker 1, 3, 6, 9, 12, or 15 years in the future (see Figures 1 and 2). Multiple device displays can be projected simultaneously, which we used to consolidate the altering measures with graphics (eg, to explain wrinkle formation). We implemented mirroring with 10 Galaxy Tab A tablets (Samsung) via Apple’s AirPlay interface using the Android app “Mirroring360” (Splashtop Inc).

In the first 10 minutes, the displayed face of one student volunteer was used to show the app’s altering features to their peer group, providing an incentive for the rest of the class to try the app.

In the following 15 minutes, students were encouraged to try the app on their own device or one of the tablet computers provided for students not owning a mobile phone or without the app. The number of provided tablet computers was calculated so the phase would take up to 12 minutes at the most, factoring in a utilization time of about 4 minutes per student. By this calculation, 25 minutes of the mirroring intervention and 10 provided tablets were sufficient to have every student within a grade of 40 pupils successfully photoaged at least once.

**Figure 3:**
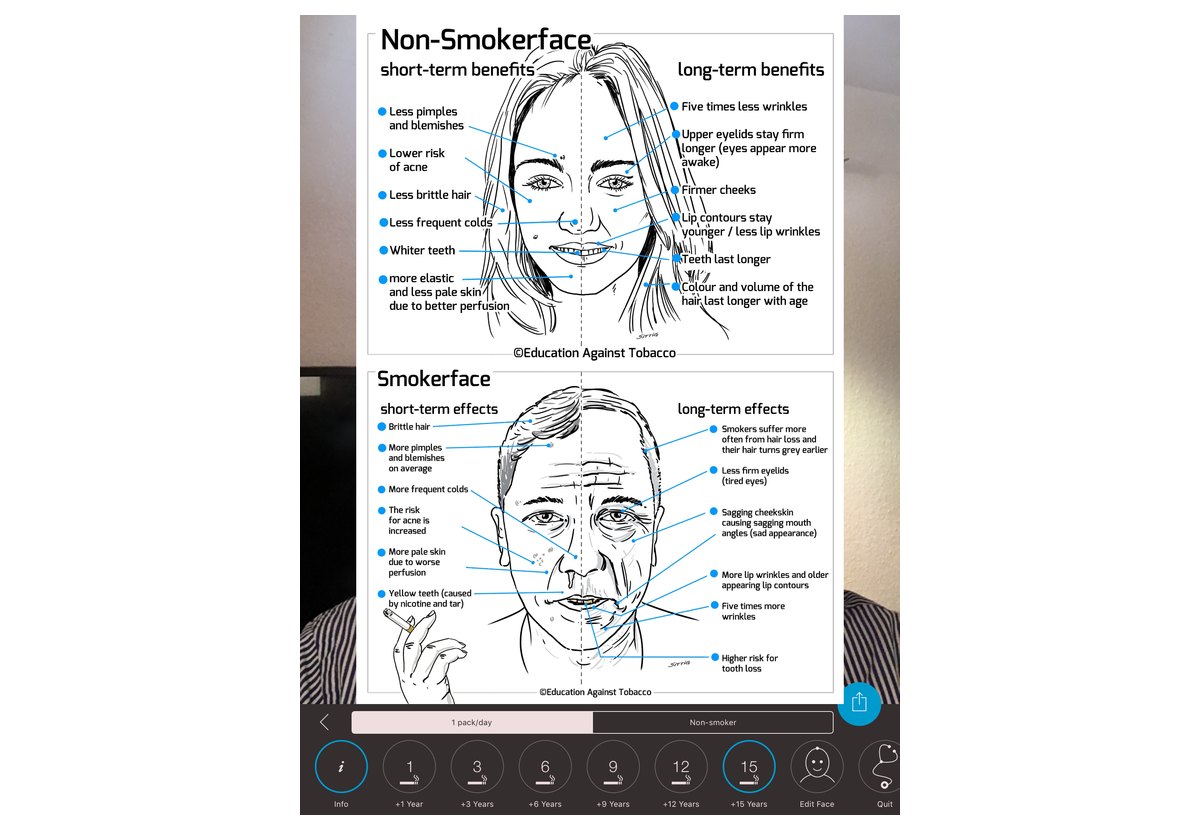
Infographic within the Smokerface app on the dermatologic short-term/long-term consequences of smoking.

**Figure 4:**
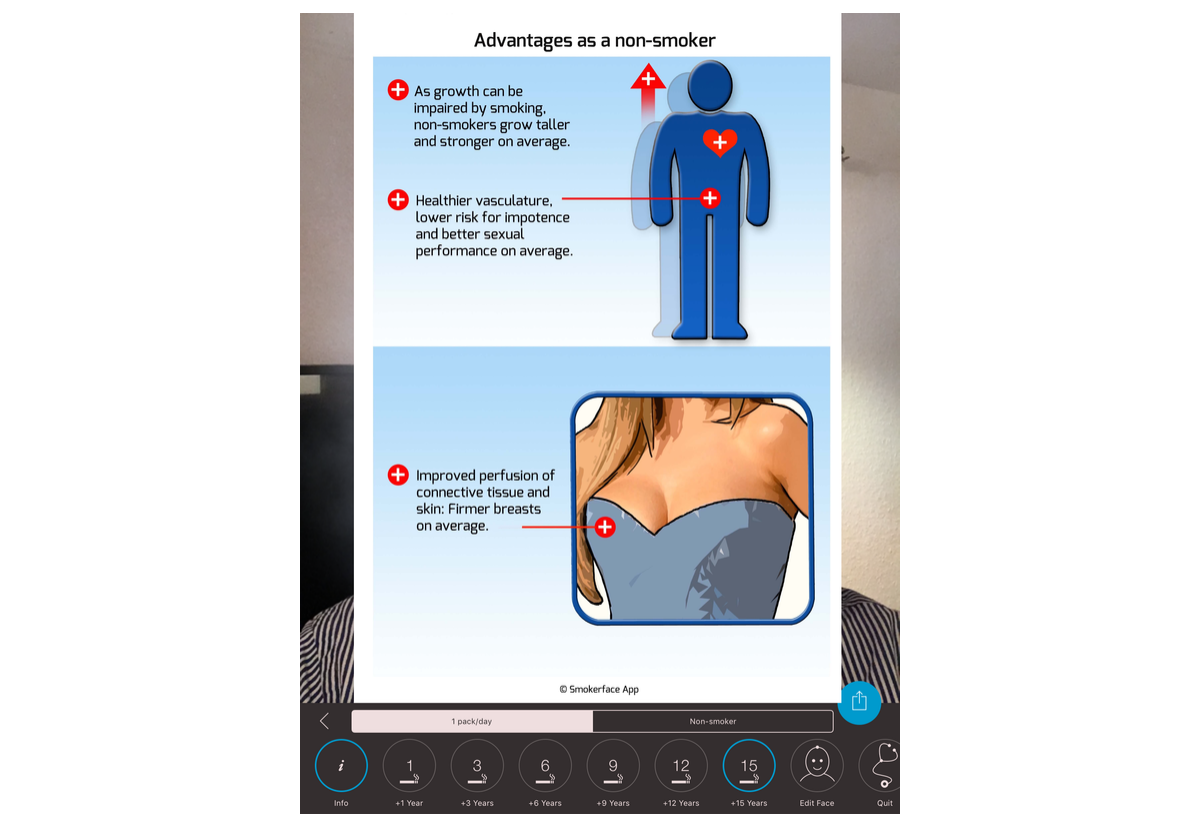
Infographic within the Smokerface app on the consequences of smoking on growth/strength and the firmness of women breasts.

This was followed by a 15-minute interactive discussion of the remaining functions of the app: facial changes, quitting via the free Smokerstop app, and impaired growth, strength, and sagginess of women’s breasts. These topics are strictly in line with the explanatory graphics within the app (Figures 3 and 4).

### Postsurvey

In the last 5 minutes of the time in the classroom, the perception of the intervention by students was measured directly after the intervention via 10 items in an anonymous survey on a 5-point Likert scale: (1) one item on change of intentions (“My 3D selfie motivates me not to smoke”), (2) two items on the perceived reactions of the peer group (“My classmates think I look better as a non-smoker” and “The reactions of my classmates motivate me not to smoke”), (3) three items on future app-use and app-sharing (“I plan to try this app again in the future,” “I want to have the Smokerface app on my phone” and “I plan to show this app to other people”), (4) four items addressing global feedback (“The intervention was fun,” “I learned new benefits of nonsmoking,” “Smokerface app motivates other people to quit smoking,” and “Smoking would have negative effects on my appearance”).

The medical students filled out a brief process evaluation consisting of six items capturing the complete implementation of the intervention as well as how the medical students perceived its effectiveness when in class.

## Results

**Figure 5:**
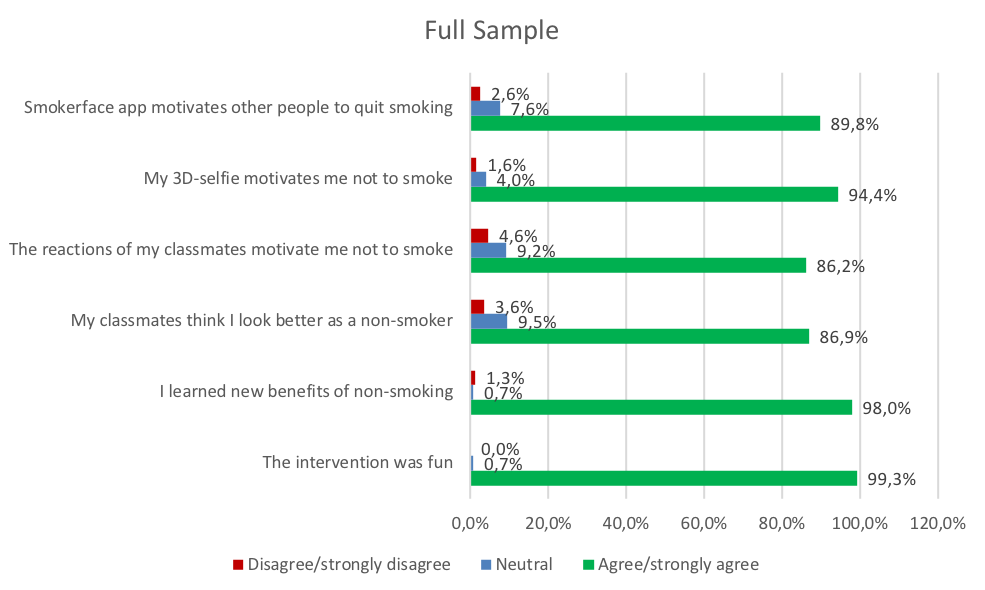
Survey results of the whole sample.

**Figure 6:**
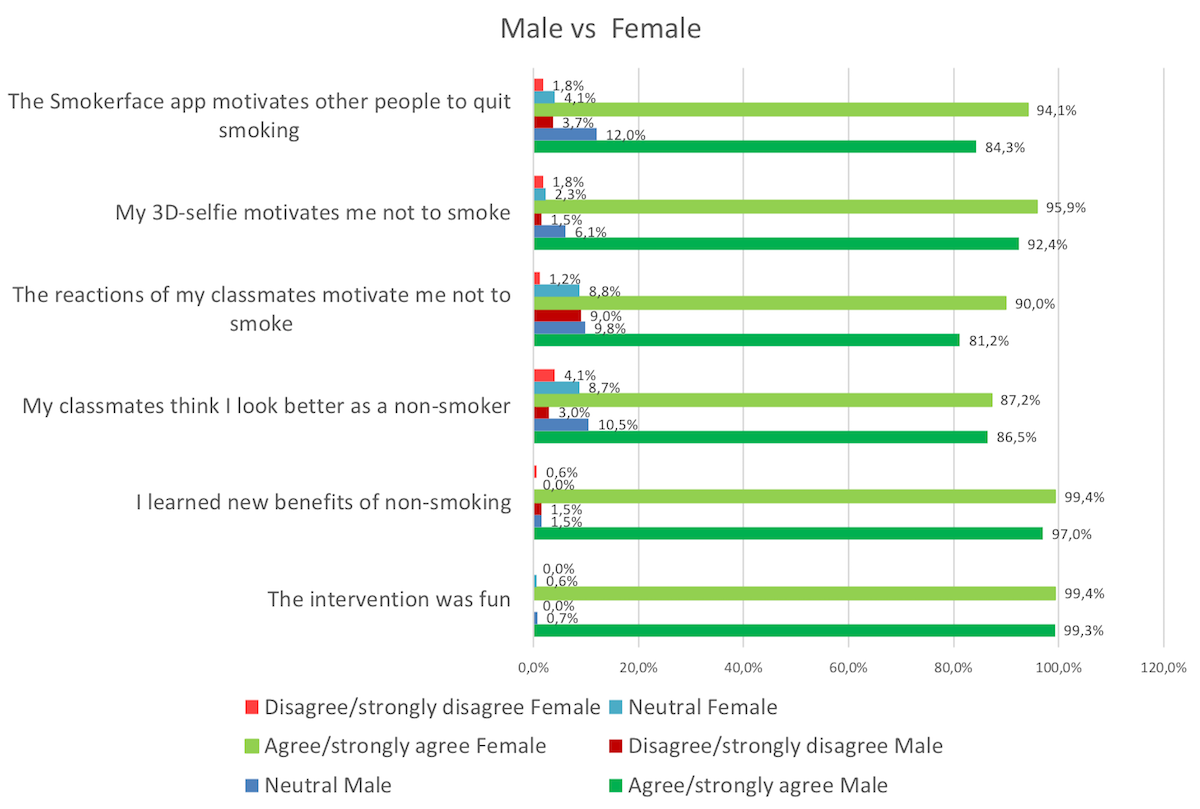
Survey results of male versus female participants.

All data were analyzed and illustrated in regards to overall perceptions of the intervention within the whole sample (Figure 5) but also to identify gender differences (Figure 6).

### Motivation Not to Smoke

We measured 94.4% (289/306) agreement on the item measuring the increase of motivation not to smoke: 94.4% agreed/fully agreed that their 3D selfie motivates them not to smoke while only 1.6% (5/306) disagreed or strongly disagreed and 4% were not sure (Figure 5). These results did not vary notably in males compared to females: in males, 92.4% (124/134) agreement and 1.5% (2/134) disagreement and, in females, 95.9% (165/172) agreement and 1.8% (3/172) disagreement (Figure 6).

### Perceived Subjective Norm During the Mirroring Intervention

The two items measuring the reactions of the peer group towards the individual selfie showed positive peer pressure to become or to remain a nonsmoker. The majority of students agreed/totally agreed that their classmates prefer them as nonsmokers (266/306, 86.9%) and that their classmates’ reaction to the 3D selfie motivates them not to smoke (264/306, 86.2%) (Figure 5). The results were similar between different genders on the first item (“My classmates think I look better as a nonsmoker”). However, females had a higher rate of agreement on the second item (“The reactions of my classmates motivate me not to smoke”): 81.2% (109/134) agreement and 9.0% (12/134) disagreement in males compared to 90.0% (155/172) agreement and 1.2% (2/172) disagreement in females (Figure 6).

### App Reuse and Sharing

We measured more than 70% agreement in all three items measuring intention to reuse or share the Smokerface app. The majority of the students expressed a desire to show the app to other people (271/306, 88.7% agreement and 10/306, 3.4% disagreement), would like to have the app on their mobile phones (215/306, 70.3% agreement and 27/306, 8.9% disagreement), and planned to try the app on themselves again later on (221/306, 72.4% agreement and 19/306, 6.2% disagreement). These results did not vary notably in males versus females.

### Global Feedback

Almost all participants expressed that they perceived the intervention as fun: 99.3% (304/306) agreement, 0.0% (0/306) disagreement, and 0.7% (2/306) neutral (Figure 5). Almost all also stated that they learned new benefits of nonsmoking: 98.0% (300/306) agreement versus 1.3% (4/306) disagreement (Figure 5). A large majority also reported that they agree/totally agree that smoking would have negative effects on their appearance (305/306, 99.7%) and that the Smokerface app motivates people to quit smoking (275/306, 89.8%). These results were similar between males and females, except for a higher female agreement on the item “Smokerface app motivates other people to quit smoking”: 84.3% (113/134) agreement and 3.7% (5/134) disagreement in males versus 94.1% (162/172) agreement and 1.8% (3/172) disagreement in females (Figure 6).

### Data Obtained From Medical Students

Our process evaluation conducted among all of the six volunteering medical students via a short questionnaire after every classroom visit revealed that 100% of the secondary school students received the mirroring intervention as outlined in the methods section. All of the medical students were able to have empathic communication with the students, regarded the intervention as enjoyable, and said it motivated them to deliver smoking cessation advice to future patients.

## Discussion

### Principal Considerations

Mobile apps are used, evaluated, and optimized in smoking cessation settings [29-52] while the number of completed randomized trials remains scarce. Mobile phone apps in school-based prevention settings present a potential new way of delivering effective interventions that remain with the pupils after the classroom visit is finished. In Brazil specifically, approximately 85% of Brazilian adolescents and young adults (10- to 24-year-olds) owns a smartphone according to the Brazilian Institute of Geography and Statistics.

### The Intervention in the Context of the Theory of Planned Behavior

The theoretical background of the participant-centered mirroring intervention includes increasing perceived self-efficacy of using the app, which has been proven to encourage repetitive use and is associated with the effectiveness of an intervention according to the theory of planned behavior [53]. Accordingly, 72.4% of the students fully agreed or agreed directly after the intervention that they wanted to use the app again on their own despite the one-time-use nature of the app and the fact that most of them had used the app at least twice already. By causing direct peer group and teacher reactions to the intervention itself, the subjective norm is affected, which also predicts adolescent smoking [53].

The theory of planned behavior identifies perceived behavioral control as the strongest predictor of smoking onset (eg, if students think they could refuse a cigarette successfully). To this end, an age-appropriate reason not to smoke was integrated into the student community by both the name of the app, “Smokerface”, and the fact that it was installed on most students’ devices. A majority (89.8%) of the students stated that the app was an appropriate tool to convince peers to quit smoking when asked after the intervention. Also, many students would refer to smokers as “smokerfaces” or stated that they did not want to be a “smokerface,” which is an age-appropriate reason to decline a cigarette if offered by a peer.

### Gender Differences

Both genders agreed in most categories, which is consistent with recent literature suggesting that appearance aspects play a major role for self-esteem in male as well as in female adolescents. While females tend to be more susceptible to appearance aspects in the past, the differences between the two sexes appear to assimilate [28,54,55].

Still, in this study a larger fraction of female participants agreed that the Smokerface app motivates other people to quit smoking (84.3% agreement in males vs 94.1% agreement in females; Figure 6) and also perceived the reactions of their classmates as a stronger motivation for abstinence (81.2% agreement in males vs 90.0% agreement in females; Figure 6), indicating a higher perception in females of subjective norms reinforcing the importance of their outward appearance.

### Limitations

Our results stem from anonymous self-reports via paper-and-pencil questionnaires filled out after the intervention. While anonymity decreases social desirability bias in self-reports, they may not be regarded as objective as externally measurable markers (eg, cotinine saliva or carbon monoxide testing). Furthermore, handing out the questionnaires after the intervention rather than before might have provoked a social desirability bias despite anonymity. In addition, cross-sectional data without a control group or follow-up cannot determine effectiveness. Thus, the authors plan to conduct a randomized trial [24].

### Conclusion

The facial-aging intervention was effective in generating an increased motivation to stay away from tobacco in Brazilian adolescents. The predictors measured indicated an even higher prospective effectiveness in southeast Brazil than in Germany (over 90% of agreement in Brazil vs over 60% of agreement in Germany on the items that measured motivation to remain abstinent) in accordance with the theory of planned behavior. Medical students are capable of complete implementation of the intervention. A randomized controlled trial measuring prospective effects in Brazil is planned as a result of this study [24].

## Appendix

Multimedia Appendix 1 Animated effect view (coughing) of the Smokerface app.
